# Optimal NaCl Medium Enhances Squalene Accumulation in *Thraustochytrium* sp. ATCC 26185 and Influences the Expression Levels of Key Metabolic Genes

**DOI:** 10.3389/fmicb.2022.900252

**Published:** 2022-05-04

**Authors:** Aiqing Zhang, Yaodong He, Biswarup Sen, Weijun Wang, Xin Wang, Guangyi Wang

**Affiliations:** ^1^Center for Marine Environmental Ecology, School of Environmental Science and Engineering, Tianjin University, Tianjin, China; ^2^Department of Microbiology, Miami University, Oxford, OH, United States; ^3^Center for Biosafety Research and Strategy, Tianjin University, Tianjin, China; ^4^Key Laboratory of Systems Bioengineering (Ministry of Education), Tianjin University, Tianjin, China

**Keywords:** thraustochytrids, NaCl, squalene, metabolism, transcriptome, optimization

## Abstract

Squalene, a natural lipid of the terpenoid family, is well-recognized for its roles in regulating cholesterol metabolism, preventing tumor development, and improving immunity. For large-scale squalene production, the unicellular marine protists—thraustochytrids—have shown great potential. However, the growth of thraustochytrids is known to be affected by salt stress, which can eventually influence the squalene content. Here, we study the effects of an optimal concentration of NaCl on the squalene content and transcriptome of *Thraustochytrium* sp. ATCC 26185. Under the optimal culture conditions (glucose, 30 g/L; yeast extract, 2.5 g/L; and NaCl, 5 g/L; 28°C), the strain yielded 67.7 mg squalene/g cell dry weight, which was significantly greater than that (5.37 mg/g) under the unoptimized conditions. NaCl was determined as the most significant (*R* = 135.24) factor for squalene production among glucose, yeast extract, and NaCl. Further comparative transcriptomics between the ATCC 26185 culture with and without NaCl addition revealed that NaCl (5 g/L) influences the expression of certain key metabolic genes, namely, *IDI*, *FAS-a*, *FAS-b*, *ALDH3*, *GS*, and *NDUFS4*. The differential expression of these genes possibly influenced the acetyl-CoA and glutamate metabolism and resulted in an increased squalene production. Through the integration of bioprocess technology and transcriptomics, this report provides the first evidence of the possible mechanisms underscoring increased squalene production by NaCl.

## Introduction

Squalene (2,6,10,15,19,23-hexamethyltetracosa-2,6,10,14,18,22-hexaene), an acyclic triterpene hydrocarbon (C_30_H_50_), is an important precursor in the biosynthesis of triterpenoids, sterol, and hopanoid (Spanova and Daum, 2011). Squalene is known for its beneficial properties, including natural antioxidant, anti-inflammatory, anti-atherosclerotic (Lou-Bonafonte et al., 2018), skin hydration (Huang et al., 2009), and adjuvant (Fox, 2009). The wide applications of squalene in cosmetics, pharmaceuticals, and nutraceutical products have led to its increasing demand in recent years. As a natural product, squalene is mainly derived from shark liver oil, but plant oils, such as olive oil (De Leonardis et al., 1998), palm oil, and rice bran oil (Huang et al., 2009) are also its common sources. However, environmental concerns of shark fishing and the low squalene content of plant oils have led to the global search for a sustainable and eco-friendly source.

Microbial sources of squalene have attracted much attention for their fast growth rates, controllable fermentation, and high production yield (Rude and Schirmer, 2009; Ghimire et al., 2016). Among the microbial sources, the unicellular, fungus-like marine, heterotrophic protists (thraustochytrids) are one of the most promising candidates for future exploration owing to their well-established fermentation technology for mass production (Fan et al., 2010). Some thraustochytrid strains have great potential for high-yield production of squalene (Hoang et al., 2014; Zhang et al., 2019). However, the squalene production in thraustochytrids can vary widely among strains and across culture conditions (Bhattacharjee et al., 2001; Chen et al., 2010). For example, the optimization of nitrogen and carbon sources has been reported to enhance squalene production (Chen et al., 2010; Fan et al., 2010; Nakazawa et al., 2012; Zhang et al., 2019). In a previous study, a drop in squalene production along with the increasing concentration of carbon and nitrogen was observed (Zhang et al., 2019). Furthermore, a low amount of NaCl in the culture medium has been reported to be beneficial for squalene production in several thraustochytrid strains (Nakazawa et al., 2012; Hoang et al., 2014; Zhang et al., 2019). Sodium has been suggested to play a role in osmotic adjustment in thraustochytrid cells without affecting cell physiology and polyunsaturated fatty acids production (Shabala et al., 2009, 2013). As culture conditions have differentiating effects on squalene production, it is important to identify the key factor that shows a significant influence on squalene metabolism.

Certain environmental factors have been reported to affect terpenoid biosynthesis through protein translation, transcription, and post-transcriptional regulation (Liu et al., 2017). For example, NaCl was an important factor of terpenoids biosynthesis (Bursy et al., 2008; Pade et al., 2016) and showed its effects on the physiological response and metabolism in some microorganisms (Klähn and Hagemann, 2011; Pade and Hagemann, 2014; Yan et al., 2015). In recent work, NaCl was found to have a role in the regulation of squalene biosynthesis in a thraustochytrid strain (Zhang et al., 2021). Thermodynamic analysis revealed that a low concentration of NaCl (5 g/L) could drive biosynthesis through enhanced energy generation. It was, therefore, important to understand further whether an optimal level of NaCl affects the transcriptome of thraustochytrids, particularly, the expression levels of genes related to the terpenoid biosynthesis.

In this study, various culture conditions were first investigated to identify the most effective factor that enhances the squalene content of a thraustochytrid strain—ATCC 26185. The influence of the most effective factor (NaCl) was then evaluated at the transcriptome level and the differential expressions of the key metabolic genes were analyzed. This study provides information about the mechanisms of NaCl-driven enhanced squalene content in thraustochytrids and key targets for future metabolic engineering efforts towards the improvement of squalene production.

## Materials and Methods

### Strain and Seed Culture

*Thraustochytrium* sp. ATCC 26185 was purchased from American Type Culture Collection (Manassas, VA, United States). The strain was maintained on an agar plate containing an SQU medium (Zhang et al., 2019). Seed culture was prepared by inoculating a single colony from the agar plate into a 150 ml Erlenmeyer flask containing 50 ml of SQU medium. The inoculated culture flask was incubated for 2 days in a shaker incubator set at 28°C and 170 rpm.

### Flask Culture Experiments

Experiments were performed in shake flasks to evaluate the growth and squalene production under different culture conditions. The seed culture (10% w/v) was transferred to a 100 ml Erlenmeyer flask containing 50 ml of fresh medium. The effects of cultivation time (1–8 days), temperature (18°C–33°C), carbon source (glucose, fructose, sucrose, glycerin, lactose, and cornstarch), nitrogen source [yeast extract, tryptone, peptone, (NH_4_)_2_SO_4_, KNO_3_, monosodium glutamate (MSG), and urea], and NaCl concentration (0–30 g/L) were investigated sequentially by the one-factor-at-a-time (OFAT) experimental design (Supplementary Table S1). In addition, experiments were carried out to study the effects of the various concentrations of the best carbon and nitrogen sources. All experiments were conducted in triplicates.

Based on the OFAT experimental results, an orthogonal design (nine-run) with glucose, yeast extract, and NaCl at three different levels was formulated. The concentrations of glucose were 25 g/L (level 1), 30 g/L (level 2), and 35 g/L (level 3), while for yeast extract and NaCl these were 2.5 g/L (level 1), 5 g/L (level 2), and 7.5 g/L (level 3). The orthogonal experiment was designed in SPSS Statistic 19 software and the results were analyzed using ANOVA. To verify the optimized squalene production, the strain was cultured in a 150 ml Erlenmeyer flask with 50 ml of the optimal medium at 28°C, 170 rpm for 3 days.

### Quantification of Cell Mass and Squalene

For the quantification of cell mass and squalene, the cells (10 ml culture) were first harvested by centrifugation at 8,000 rpm for 5 min and then washed twice with double distilled water and lyophilized for 48 h in a freeze dryer (Christ, Germany). These lyophilized cells were stored at −80°C until further processing. The cell mass and squalene concentration of the lyophilized cells were measured by the gravimetric method and previously described method (Hoang et al., 2014), respectively. Squalene was extracted from the lyophilized cells after saponification. For saponification, a mixture of 1.5 ml chloroform:methanol (2:1, v/v) was added to the lyophilized cells and vortexed for 1 min. The cells were then transferred into a glass tube containing 4 ml of 15% (w/v) KOH-methanol: H_2_O (4,1, v/v), and the tube was incubated at 60°C in a water bath for 2 h. After cooling down the tube to room temperature, the unsaponifiable fraction containing squalene was extracted in 2 ml n-hexane. The upper hexane layer was used for squalene quantification by GC (Agilent 7890B, United States) equipped with a DB-WAX column (60 m × 320 μm × 0.15 μm). The temperature of the flame ionization detector port was set to 250°C and that of the oven was held at 50°C for 1 min. The oven temperature was first increased to 180°C at the rate of 20°C/min, then to 250°C at the rate of 15°C/min, and finally, hold down at 250°C for 13 min.

### Transcriptome Analysis

To understand the possible mechanisms of how NaCl affects squalene biosynthesis, the transcriptome of the strain ATCC 26185 was analyzed between the cultures grown in optimal medium with 0 g/L (NaCl-0) and 5 g/L (NaCl-5) of NaCl. Triplicate culture samples were analyzed for each group. Cells were harvested from the 54-h culture sample and their total RNA was extracted for mRNA isolation. The isolated mRNA was then reverse transcribed to cDNA for next-generation RNA sequencing (RNA-Seq). RNA-Seq was performed by Biomarker Technologies Co. Ltd. (Beijing, China). The clustering of the index-coded samples was performed on a cBot Cluster Generation System using TruSeq PE Cluster Kit v3-cBot-HS (Illumina) according to the manufacturer’s instructions. After cluster generation, the library preparations were sequenced on an Illumina Hiseq 2000 platform and paired-end reads were generated. The raw reads were trimmed and filtered using the Trimmomatic platform (Bolger et al., 2014). The resulting clean reads were then mapped *de novo*. A total of 30,490 transcripts with an average length of 1360.75 and 22,869 unigenes with an average length of 1254.13 were obtained after assembly using the Trinity platform (Grabherr et al., 2011; Supplementary Table S2).

The gene expression was quantified using the RSEM software (Li and Dewey, 2011). Benjamin Hochberg method was used to correct the significance value of *p* obtained from the original hypothesis test, and the corrected value of *p*, namely false discovery rate (FDR), was used as the key index for differential gene expression (DGE) analysis. An FDR ≤ 0.01 and the log_2_ fold change (log_2_FC) ≥ 2 were used for screening differentially expressed genes (DEGs). Enrichment factor was used to analyze the enrichment degree of the pathway, and Fisher’s exact test was used to calculate the enrichment significance. Gene function was annotated based on the following databases: NR (NCBI non-redundant protein sequences), Pfam, KOG/COG/eggNOG, Swiss-Prot, KEGG, nr, and GO. A total of 10,357 unigenes were annotated on these databases. GO annotation identified 2,763 unigenes, 44 processes, and 123 DEGs related to biological process, cellular component, and molecular function (Supplementary Figure S1). Of the total DEGs, 63, 56, and 57 were functionally associated with the cellular process, cellular component, and catalytic activity, respectively. No significantly enriched KEGG pathways were found (Supplementary Figure S2).

### Statistical Analysis

The group means of biomass and squalene concentration and yield were statistically analyzed at the alpha level of 0.05 using ANOVA. When the ANOVA *F* test was significant, the specific differences between the group means were analyzed using the Duncan *post hoc* test. All statistical tests were conducted in SPSS Statistic 19 software.

## Results and Discussion

### Effects of Culture Conditions on Squalene Production

**Figure 1:**
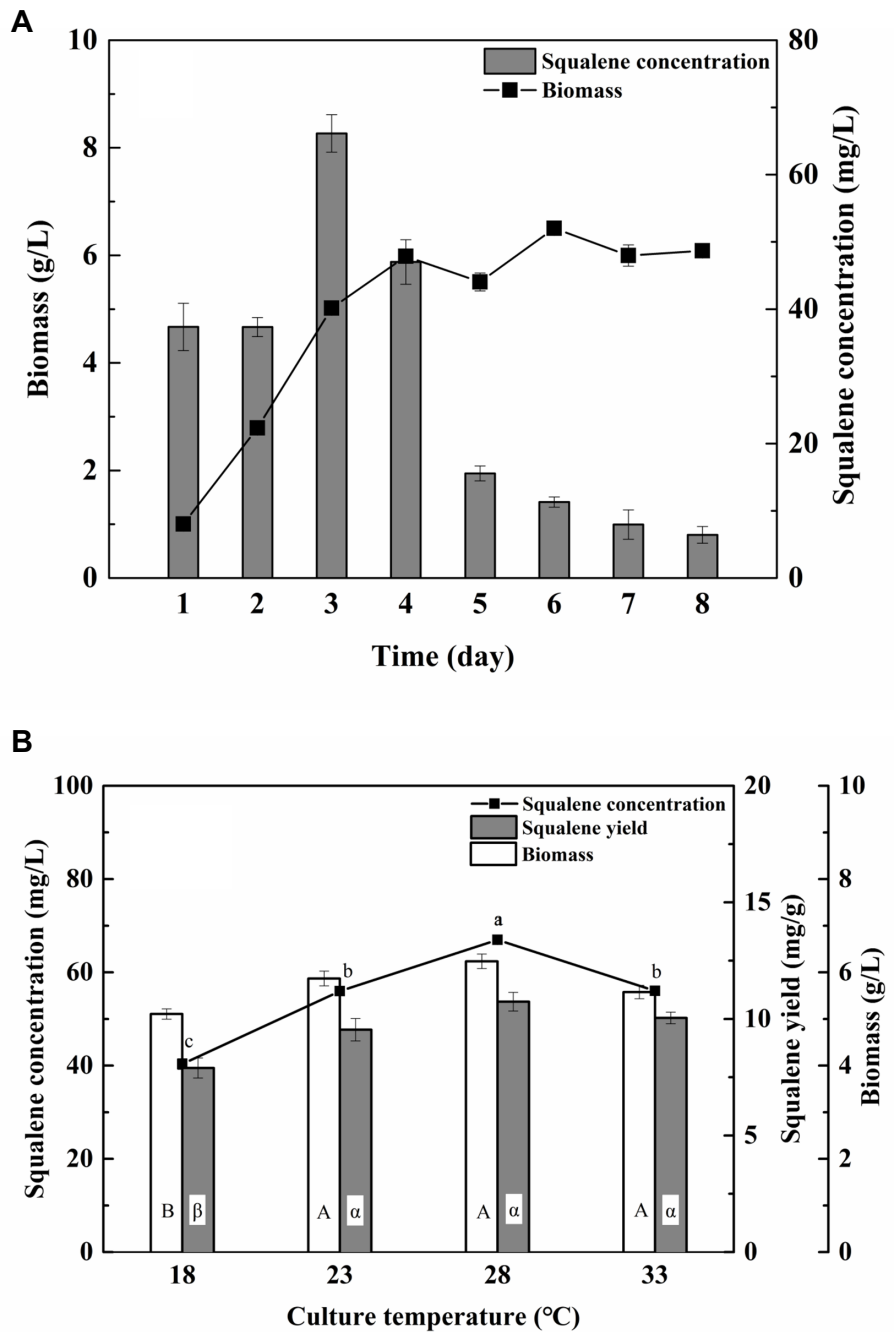
Effect of **(A)** incubation time and **(B)** incubation temperature on the biomass and squalene production in ATCC 26185 culture. (a–c): ANOVA of squalene concentration (mg/L), (α, β): ANOVA of squalene yield (mg/g), and (A,B): ANOVA of biomass (g/L).

The flask culture experiments to study the effect of the incubation period and culture temperature revealed 3 days and 28°C as the optima for maximum yield of squalene (66.97 mg/L and 5.37 mg/g; Figure 1). The concentration of squalene decreased significantly after 3 days of incubation despite high and uniform biomass (Figure 1A). Similar effects of incubation period on biomass and squalene production have been reported previously (Fan et al., 2010; Zhang et al., 2019), suggesting rapid oxidation of squalene to squalene epoxide and/or its utilization as a carbon source (Bhattacharjee et al., 2001). Furthermore, the biomass and yield of squalene did not change significantly between 23 and 33°C (Figure 1B). Therefore, 3 days of incubation and 28°C were used in further optimization experiments.

**Figure 2:**
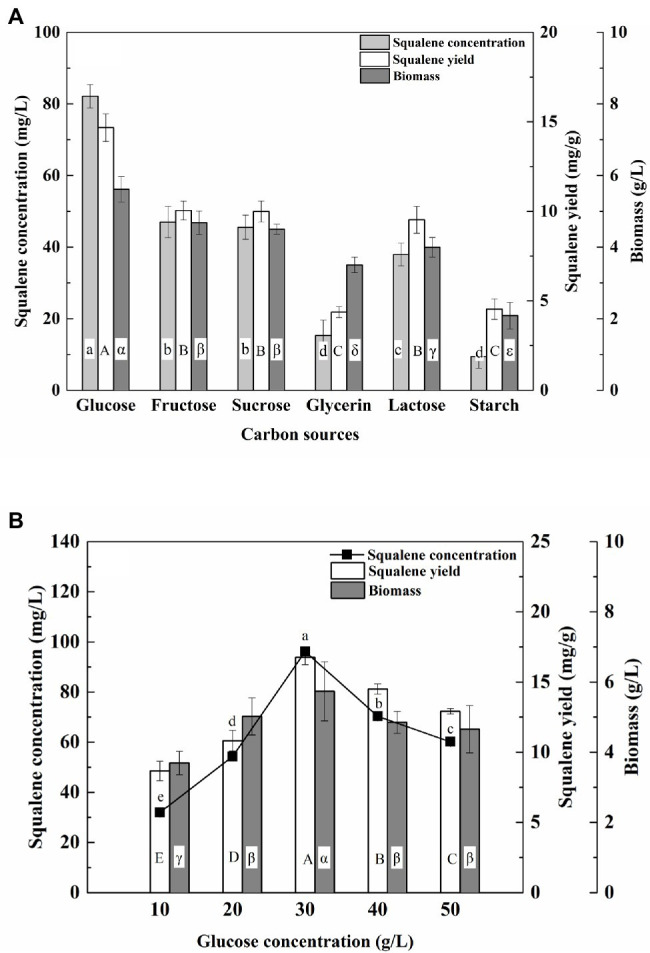
Effect of **(A)** carbon source and **(B)** glucose concentration on the biomass and squalene production in ATCC 26185 culture. (a–e): ANOVA of squalene concentration (mg/L), (A–E): ANOVA of squalene yield (mg/g), and (α–ε): ANOVA of biomass (g/L).

Among the tested carbon sources, glucose was the best for high biomass (5.62 g/L) and squalene production (82.14 mg/L, 5.62 mg/g; Figure 2A). Furthermore, the concentration and yield of squalene increased along with the glucose concentration range of 10–30 g/L and decreased when the glucose concentration was more than 30 g/L (Figure 2B). A similar pattern of squalene production along with an increasing concentration of glucose was observed for *Aurantiochytrium* sp. TWZ-97 (Zhang et al., 2019). The drop in squalene production with glucose concentration above 30 g/L could be due to the high osmotic stress generated by the excess glucose concentration (Singh et al., 2016). With 30 g/L of glucose, the ATCC 26185 culture produced the maximum biomass (5.74 g/L) and squalene (96.15 mg/L, 16.76 mg/g). Similar glucose concentration also yielded high squalene production in the TWZ-97 strain (Zhang et al., 2019).

**Figure 3:**
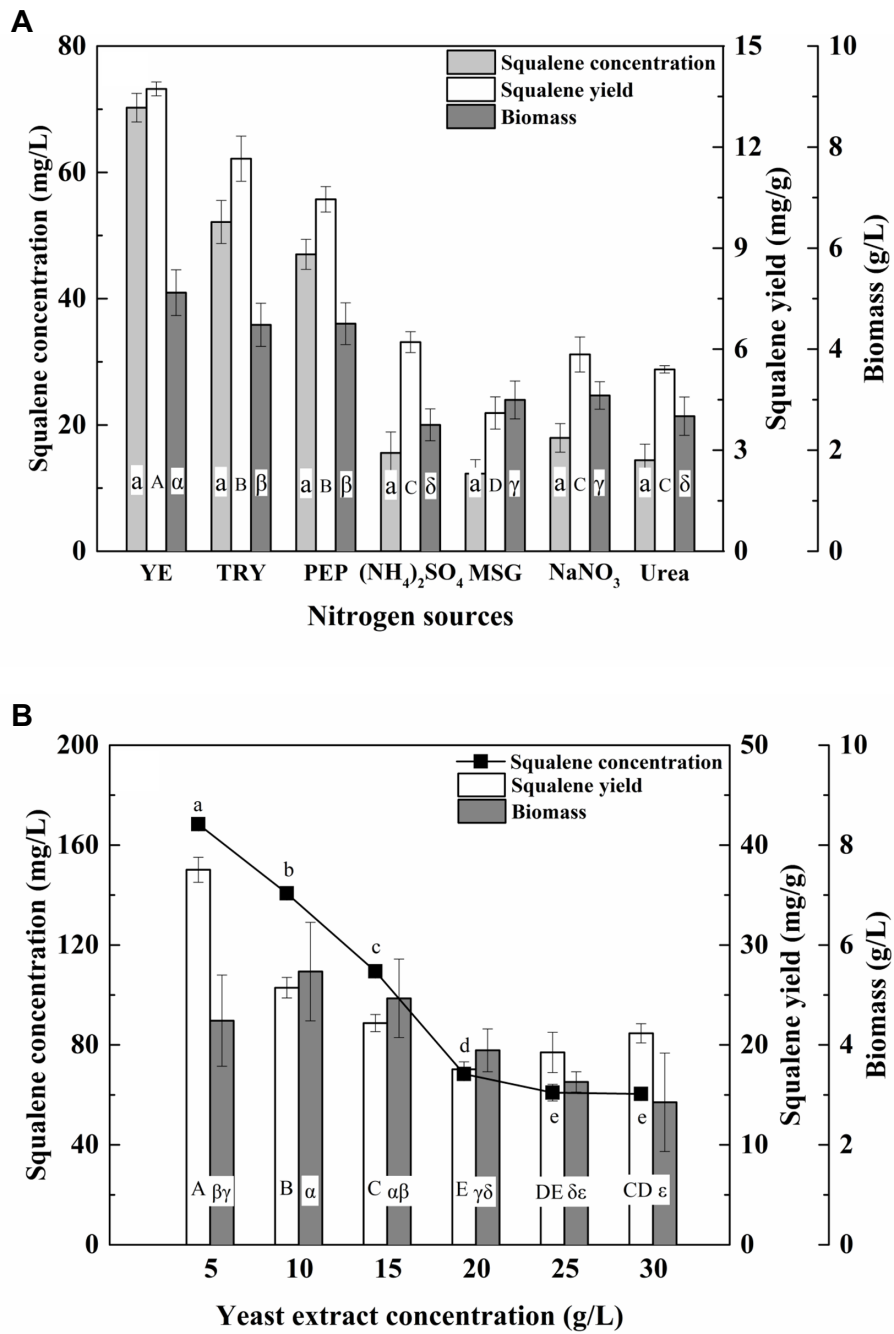
Effect of **(A)** nitrogen source and **(B)** yeast extract concentration on the biomass and squalene production in ATCC 26185 culture. YE, yeast extract; TRY, tryptone; PEP, peptone; MSG, sodium glutamate. (a–e): ANOVA of squalene concentration (mg/L), (A–E): ANOVA of squalene yield (mg/g), and (α–ε): ANOVA of biomass (g/L).

Nitrogen is an important factor that affects the growth of thraustochytrids and their squalene production (Chen et al., 2010). In this study, the ATCC 26185 culture produced significantly (*p* < 0.05) higher biomass and squalene with tryptone, peptone, or yeast extract compared with the other nitrogen sources (Figure 3A). Particularly, with the addition of yeast extract, the culture achieved the maximum biomass (5.12 g/L) and squalene concentration (70.25 mg/L). Furthermore, a low concentration of yeast extract (5 g/L) produced the maximum squalene (168.34 mg/L, 37.53 mg/g; Figure 3B), while the addition of a large concentration of yeast extract (10–30 g/L) reduced the squalene production. Previous studies have reported yeast extract as the best nitrogen source for squalene production in thraustochytrids (Wang et al., 2018; Zhang et al., 2019). However, under excess nitrogen the productivity of fatty acids has been found to drop (Heggeset et al., 2019). Our study demonstrates that an excess of yeast extract (>5 g/L) can also significantly lower the squalene production in ATCC 26185 strain.

**Figure 4:**
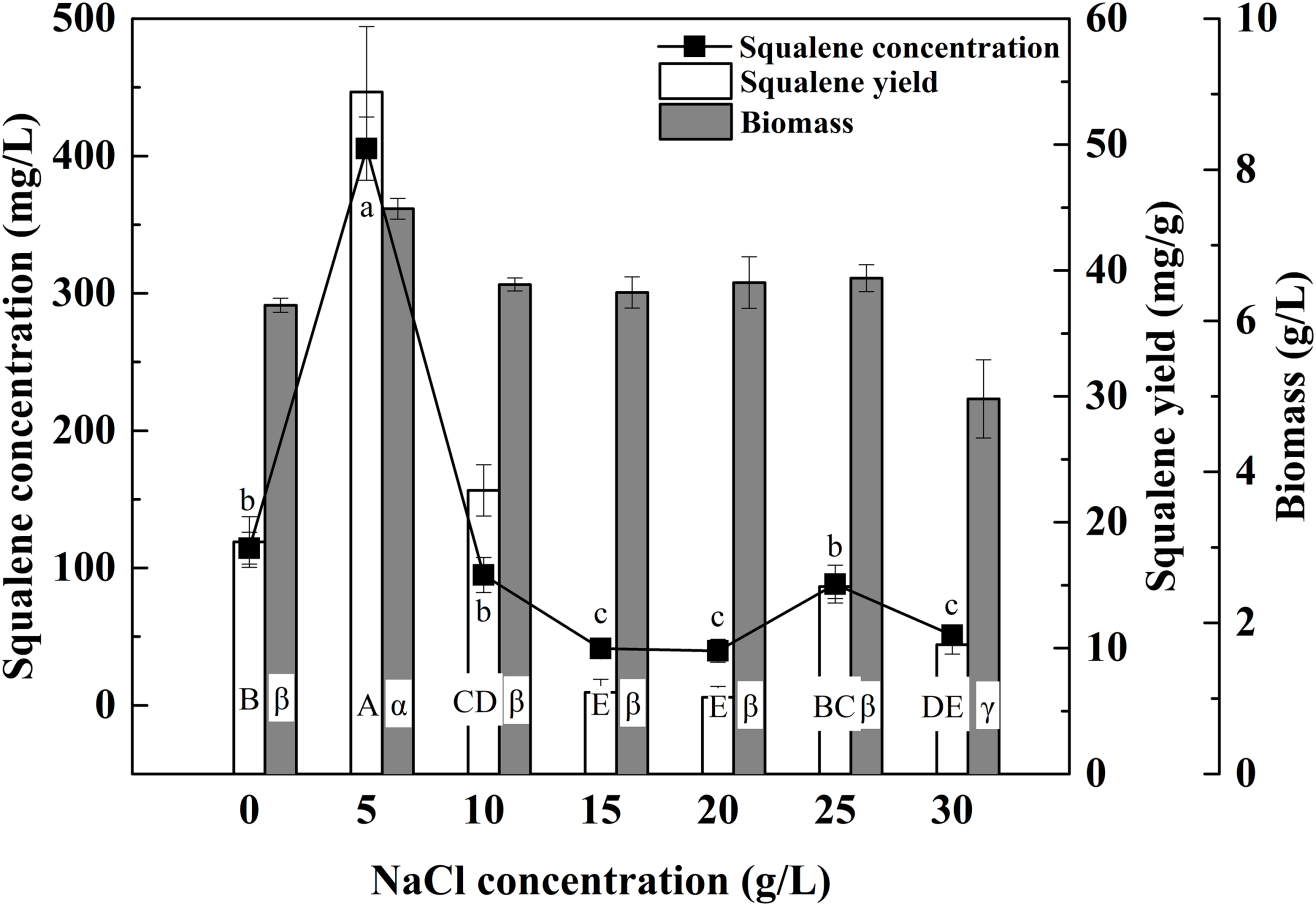
Effect of NaCl concentration on the biomass and squalene production in ATCC 26185 culture, (a–c): ANOVA of squalene concentration (mg/L), (A–E): ANOVA of squalene yield (mg/g), and (α–γ): ANOVA of biomass (g/L).

The ATCC 26185 culture produced the maximum biomass with 5 g/L of NaCl while it produced the minimum biomass with 30 g/L of NaCl (Figure 4). These observations are consistent with the previous reports on the effect of seawater concentration on the growth of *Aurantiochytrium* strains, namely 18W-13a (Nakazawa et al., 2012), mh0186 (Nagano et al., 2009), and SR21 (Yokochi et al., 1998). In addition, the culture showed a considerable amount of biomass without any addition of NaCl, which suggested that Na^+^ ions are not essential for the growth of the ATCC 26185 strain. In the presence of other ions such as Cl^−^ and K^+^ in the medium, thraustochytrid cells undergo osmotic adjustments upon hypoosmotic stress (Shabala et al., 2009). As the SQU medium contained both these ions (Supplementary Table S1), the requirement for Na^+^ in osmotic adjustment was perhaps not vital for the growth of the ATCC 26185 strain. Furthermore, an optimal concentration of NaCl (5 g/L) was found to boost the squalene concentration and yield by up to 405.41 mg/L and 54.18 mg/g, respectively. These findings are in agreement with the previous study where an optimal concentration of NaCl (7.5 g/L) was reported to enhance the squalene production by a thraustochytrid strain TWZ-97 (Zhang et al., 2019). More importantly, the major advantage of an optimal NaCl concentration is that it is favorable to the biotechnological industry because it can reduce the corrosive effects of chloride on fermentation equipment (Barclay, 2002).

Overall, the OFAT experimental results indicate that culture conditions can significantly influence the growth and squalene production of the ATCC 26185 strain. The addition of glucose (30 g/L), yeast extract (5 g/L), NaCl (5 g/L), and cultivation at 28°C for 3 days are the most favorable culture conditions to maximize the squalene production. Although the achieved squalene yield was somewhat lower compared with that of the reported high-yielding thraustochytrids strains (Patel et al., 2019, 2020), our study provides the first comprehensive report on the effects of a wide range of culture conditions on squalene production by ATCC 26185 strain.

### Optimal Conditions to Maximize Squalene Production

To further determine the best combination of glucose, yeast extract, and NaCl levels for a maximum yield of squalene, an orthogonal experiment [L_9_(3^3^)] was performed (Tables 1, 2). The mean analysis revealed that the optimal concentrations of glucose, yeast extract, and NaCl for squalene production were 30, 2.5, and 5 g/L, respectively (Supplementary Table S2). Under these optimal conditions, the ATCC 26185 culture yielded 456.3 mg/L of squalene. Further batch experiments were conducted for verification of the results obtained with the orthogonal experimental design. The verification experiments showed 2.9 g/L of biomass, 160 mg/L of squalene, and 55.2 mg/g squalene yield at 72 h of fermentation under optimized culture conditions with 0 g/L NaCl (Supplementary Figure S3A). Conversely, the biomass, squalene content, and squalene yield were 6.3 g/L, 428 mg/L, and 67.7 mg/g, respectively, under optimized culture conditions with 5 g/L (Supplementary Figure S3B). Overall, the optimal culture conditions described in our study suggest a significant improvement in the cell growth of the ATCC 26185 strain and its squalene production.

**Table 1:** Orthogonal experimental design and analysis for glucose, yeast extract, and NaCl as the factors.

Runs	Factor levels			Squalene (mg/L)^#^
	Glucose	Yeast extract	NaCl	
1	1	1	1	285.8
2	2	2	1	300.7
3	3	3	1	263.6
4	2	1	2	456.3
5	3	2	2	364.7
6	1	3	2	371.2
7	3	1	3	282.3
8	1	2	3	200.4
9	2	3	3	303.8
R	67.81	52.87	135.24	

# Squalene concentration at 72 h of fermentation is provided.

**Table 2:** Factors and respective levels of the orthogonal experimental design.

Levels	Factors		
	A NaCl (g/L)	B Yeast extract (g/L)	C Glucose (g/L)
1	2.5	2.5	25
2	5	5	30
3	7.5	7.5	35

The orthogonal experimental results also revealed glucose and NaCl as the significant (*p* < 0.05) factors affecting the concentration and yield of squalene (Table 3). However, NaCl showed the highest R level (Table 1) and significance (*p* = 0.008), which suggested that between glucose and NaCl, the latter was the most influential factor in improving the squalene production. Our results support the importance of an optimal NaCl medium in maximizing the growth of thraustochytrids (Shabala et al., 2009) and also suggest its possible role in squalene biosynthesis.

**Table 3:** ANOVA output of the orthogonal experiment with glucose, yeast extract, and NaCl as the factors.

Factors	SS	df	MS	*F* value	*p* value
Modified model	43360.79	6	7226.80	56.43	0.018
Intercept	889123.27	1	889123.27	6942.599	0
Glucose	7417.98	2	3709.00	28.96	0.034
Yeast extract	4202.44	2	2101.22	16.27	0.058
NaCl	31745.72	2	15872.86	122.87	0.008
Error	258.37	2	129.19		

### Differential Gene Expression Under Optimal NaCl Condition

In this study, a total of 167 DEGs were annotated on the KEGG database with no significantly enriched KEGG pathways. However, six key metabolic genes were identified, namely, *IDI*, *FAS-a*, *FAS-b*, *ALDH3*, *GS*, and *NDUFS4*, which were differentially (FDR ≤ 0.01, absolute value of log_2_FC ≥ 1) expressed between the NaCl-0 and NaCl-5 groups (Table 4). The *IDI* gene of the mevalonate pathway was significantly upregulated in the NaCl-5 group with an expression level 2.47-fold that of the NaCl-0 group. *IDI* gene encodes isopentenyl-diphosphate isomerase, which catalyzes the conversion of isopentenyl diphosphate into dimethylallyl diphosphate—the bottleneck step in terpenoid biosynthesis (Berthelot et al., 2012). The co-overexpression of isopentenyl-diphosphate isomerase and triterpene synthase has been proved to be an effective strategy for increasing triterpene production (Choi et al., 2016).

**Table 4:** Expression level and fold change of DEGs (FDR ≤ 0.01) in ATCC 26185 strain cultivated with and without NaCl (5 g/L).

Gene	KO	Expression quantity (RPKM)		Fold change	Log_2_ (fold change)
		NaCl-0	NaCl-5		
*IDI*	K01823	88.49	218.97	2.47	1.2695
*FAS*-α	K00667	258.24	105.00	0.41	−1.2823
*FAS*-β	K00668	153.87	57.45	0.37	−1.4093
*ALDH3*	K00129	30.54	69.84	2.29	1.1746
*GS*	K01915	36.77	17.99	0.49	−1.0203
*NDUFS4*	K03937	174.16	362.35	2.08	1.0328

*FAS-a* and *FAS-b* genes involved in fatty acid biosynthesis were significantly downregulated in the NaCl-5 group by 0.46-fold and 0.37-fold, respectively (Table 4). These genes encode the fatty acid synthase subunits α (FAS-α) and β (FAS-β), respectively. Therefore, the significant downregulation of these two genes could reduce the consumption of acetyl-CoA in the fatty acid biosynthesis pathway, which may be beneficial to squalene biosynthesis. Furthermore, the *ALDH3* gene was significantly upregulated by 2.29-fold in the optimal NaCl medium. The product of the *ALDH3* gene catalyzes the generation of acetyl-CoA from acetaldehyde (Eglinton et al., 2002). Previous studies have reported that acetyl-CoA acts as the precursor for both fatty acid and squalene biosynthetic pathways (Morabito et al., 2019). As a precursor for squalene biosynthesis, the acetyl-CoA pool has an essential role in the squalene production by oleaginous microorganisms (Huang et al., 2018; Liu et al., 2020). Our findings suggest that the upregulation of genes involved in the biosynthesis of acetyl-CoA in an optimal NaCl medium could have a possible role in the improved squalene accumulation in ATCC 26185 strain.

The *GS* gene expression in the NaCl-5 group was found to be significantly downregulated by 0.49-fold (Table 4). *GS* gene encodes glutamine synthetase, which catalyzes the condensation of glutamate to glutamine. The low expression level of this gene could increase the glutamate pool, which can enter the TCA cycle and contribute to ATP production eventually. In addition, as a compatible solute for osmotic adjustment, the possible glutamate build-up may contribute to osmotic stress resistance (Kang and Hwang, 2018; Zhang et al., 2021).

The expression level of *NDUFS4* gene in the NaCl-5 group was 2.08-fold that of the NaCl-0 group (Table 4). This gene encodes NADH dehydrogenase, which belongs to proton translocation complex I and is an important component of the electron transport chain. The increased expression level of the *NDUFS4* gene perhaps indicates that NaCl can influence ATP generation in the ATCC 26185 strain. Previous studies have shown that the enhancement of ATP synthesis contributes to greater squalene synthesis since the mevalonate pathway contains three ATP-dependent phosphorylation and decarboxylation steps (Yang et al., 2016). Moreover, the accumulation of ATP may provide a strong thermodynamic driving force for the mevalonate pathway constrained by the ACAT-catalyzed reaction (Zhang et al., 2021), eventually contributing toward terpene biosynthesis in thraustochytrids.

Overall, the transcriptomics data possibly suggest that an optimal NaCl medium may elevate the fluxes of acetyl-CoA and compatible solutes, which eventually contribute toward squalene biosynthesis and osmotic adjustments in thraustochytrids.

Nevertheless, fluxomics is essential to provide direct evidence to reveal the metabolic fluxes in an optimal NaCl medium. Interestingly, the identification of key genes in our study would provide valuable information for future metabolic engineering of thraustochytrids strains toward better squalene production.

## Conclusion

In this study, ATCC 26185 strain showed a significant increase in its squalene content under optimized culture conditions. Although glucose and NaCl were both significant factors for squalene production in ATCC 26185 culture, the role of NaCl was pivotal. Furthermore, comparative transcriptomics revealed that an optimal NaCl medium might induce acetyl-CoA flux, resulting in increased squalene biosynthesis. In addition, an optimal NaCl medium also downregulates the metabolic gene that converts glutamate to glutamine, thereby increasing the glutamate pool in the cells. The increased glutamate level could be one of the mechanisms of osmotic adjustments in the ATCC 26185 strain. This study demonstrated that an optimal NaCl medium could influence some key metabolic genes, which eventually result in the increased biomass and squalene content in the cells of the ATCC 26185 strain.

## Data Availability Statement

The datasets presented in this study can be found in online repositories. The names of the repository/repositories and accession number(s) can be found at: https://www.ncbi.nlm.nih.gov/, SAMN23341422 to SAMN23341427.

## Author Contributions

AZ and GW: conceptualization. AZ, WW, and BS: methodology. AZ and YH: validation. AZ and BS: formal analysis, data curation, and visualization. AZ and WW: investigation. YH and GW: resources. AZ, BS, and GW: writing—original draft preparation and writing—review and editing. GW and XW: supervision. YH: project administration. GW: funding acquisition. All authors contributed to the article and approved the submitted version.

## Funding

This work was partially supported by China Ocean Mineral Resources R&D Association (COMRA) Program (DY135-B2-09), National Science Foundation of China (32170063), Think Tank United Foundation of Qingdao Marine Engineering and Technology (201707071001), Pilot National Laboratory for Marine Science and Technology (Qingdao), and Marine Biology and Biotechnology Laboratory 2018 Open Foundation Program, granted to GW.

## Conflict of Interest

The authors declare that the research was conducted in the absence of any commercial or financial relationships that could be construed as a potential conflict of interest.

## Publisher’s Note

All claims expressed in this article are solely those of the authors and do not necessarily represent those of their affiliated organizations, or those of the publisher, the editors and the reviewers. Any product that may be evaluated in this article, or claim that may be made by its manufacturer, is not guaranteed or endorsed by the publisher.

## Supplementary Material

The Supplementary Material for this article can be found online at: https://www.frontiersin.org/articles/10.3389/fmicb.2022.900252/full#supplementary-material

## References

[ref1] Barclay W. R. B. (2002). Reducing corrosion in a fermentor by providing sodium with a non-chloride sodium salt. United States patent application 6410281.

[ref2] Berthelot K. Estevez Y. Deffieux A. Peruch F. (2012). Isopentenyl diphosphate isomerase: a checkpoint to isoprenoid biosynthesis. Biochimie 94, 1621–1634. doi: 10.1016/j.biochi.2012.03.021, PMID: 22503704

[ref3] Bhattacharjee P. Shukla V. B. Singhal R. S. Kulkarni P. R. (2001). Studies on fermentative production of squalene. World J. Microbiol. Biotechnol. 17, 811–816. doi: 10.1023/A:1013573912952

[ref4] Bolger A. M. Lohse M. Usadel B. (2014). Trimmomatic: a flexible trimmer for Illumina sequence data. Bioinformatics 30, 2114–2120. doi: 10.1093/bioinformatics/btu170, PMID: 24695404 PMC4103590

[ref5] Bursy J. Kuhlmann A. U. Pittelkow M. Hartmann H. Jebbar M. Pierik A. J.. (2008). Synthesis and uptake of the compatible solutes ectoine and 5-hydroxyectoine by *Streptomyces coelicolor* A3(2) in response to salt and heat stresses. Appl. Environ. Microbiol. 74, 7286–7296. doi: 10.1128/aem.00768-08, PMID: 18849444 PMC2592907

[ref6] Chen G. Fan K. W. Lu F. P. Li Q. Aki T. Chen F.. (2010). Optimization of nitrogen source for enhanced production of squalene from thraustochytrid *Aurantiochytrium* sp. New Biotechnol. 27, 382–389. doi: 10.1016/j.nbt.2010.04.005, PMID: 20412873

[ref7] Choi S. Y. Lee H. J. Choi J. Kim J. Sim S. J. Um Y.. (2016). Photosynthetic conversion of CO2 to farnesyl diphosphate-derived phytochemicals (amorpha-4,11-diene and squalene) by engineered cyanobacteria. Biotechnol. Biofuels 9:202. doi: 10.1186/s13068-016-0617-8, PMID: 27688805 PMC5034544

[ref8] De Leonardis A. Macciola V. De Felice M. A. (1998). Rapid determination of squalene in virgin olive oils using gas-liquid chromatography. Italian J. Food Sci. 10, 75–80.

[ref9] Eglinton J. M. Heinrich A. J. Pollnitz A. P. Langridge P. Henschke P. A. de Barros Lopes M. (2002). Decreasing acetic acid accumulation by a glycerol overproducing strain of *Saccharomyces cerevisiae* by deleting the ALD6 aldehyde dehydrogenase gene. Yeast 19, 295–301. doi: 10.1002/yea.834, PMID: 11870853

[ref10] Fan K. W. Aki T. Chen F. Jiang Y. (2010). Enhanced production of squalene in the thraustochytrid *Aurantiochytrium mangrovei* by medium optimization and treatment with terbinafine. World J. Microbiol. Biotechnol. 26, 1303–1309. doi: 10.1007/s11274-009-0301-2, PMID: 24026934

[ref11] Fox C. B. (2009). Squalene emulsions for parenteral vaccine and drug delivery. Molecules 14, 3286–3312. doi: 10.3390/molecules14093286, PMID: 19783926 PMC6254918

[ref12] Ghimire G. P. Thuan N. H. Koirala N. Sohng J. K. (2016). Advances in biochemistry and microbial production of squalene and its derivatives. J. Microbiol. Biotechnol. 26, 441–451. doi: 10.4014/jmb.1510.10039, PMID: 26643964

[ref13] Grabherr M. G. Haas B. J. Yassour M. Levin J. Z. Thompson D. A. Amit I.. (2011). Full-length transcriptome assembly from RNA-Seq data without a reference genome. Nat. Biotechnol. 29, 644–652. doi: 10.1038/nbt.1883, PMID: 21572440 PMC3571712

[ref14] Heggeset T. M. B. Ertesvåg H. Liu B. Ellingsen T. E. Vadstein O. Aasen I. M. (2019). Lipid and DHA-production in *Aurantiochytrium* sp.—responses to nitrogen starvation and oxygen limitation revealed by analyses of production kinetics and global transcriptomes. Sci. Rep. 9:19470. doi: 10.1038/s41598-019-55902-4, PMID: 31857635 PMC6923395

[ref15] Hoang M. H. Ha N. C. Thom le T. Tam L. T. Anh H. T. Thu N. T.. (2014). Extraction of squalene as value-added product from the residual biomass of *Schizochytrium mangrovei* PQ6 during biodiesel producing process. J. Biosci. Bioeng. 118, 632–639. doi: 10.1016/j.jbiosc.2014.05.015, PMID: 24973317

[ref16] Huang Y. Y. Jian X. X. Lv Y. B. Nian K. Q. Gao Q. Chen J.. (2018). Enhanced squalene biosynthesis in *Yarrowia lipolytica* based on metabolically engineered acetyl-CoA metabolism. J. Biotechnol. 281, 106–114. doi: 10.1016/j.jbiotec.2018.07.001, PMID: 29986837

[ref17] Huang Z. R. Lin Y. K. Fang J. Y. (2009). Biological and pharmacological activities of squalene and related compounds: potential uses in cosmetic dermatology. Molecules 14, 540–554. doi: 10.3390/molecules14010540, PMID: 19169201 PMC6253993

[ref18] Kang Y. Hwang I. (2018). Glutamate uptake is important for osmoregulation and survival in the rice pathogen *Burkholderia glumae*. PLoS One 13:e0190431. doi: 10.1371/journal.pone.0190431, PMID: 29293672 PMC5749808

[ref19] Klähn S. Hagemann M. (2011). Compatible solute biosynthesis in cyanobacteria. Environ. Microbiol. 13, 551–562. doi: 10.1111/j.1462-2920.2010.02366.x, PMID: 21054739

[ref20] Li B. Dewey C. N. (2011). RSEM: accurate transcript quantification from RNA-Seq data with or without a reference genome. BMC Bioinformatics 12:323. doi: 10.1186/1471-2105-12-323, PMID: 21816040 PMC3163565

[ref21] Liu W. Wang H. Chen Y. Zhu S. Chen M. Lan X.. (2017). Cold stress improves the production of artemisinin depending on the increase in endogenous jasmonate. Biotechnol. Appl. Biochem. 64, 305–314. doi: 10.1002/bab.1493, PMID: 26988377

[ref22] Liu H. Wang F. Deng L. Xu P. (2020). Genetic and bioprocess engineering to improve squalene production in *Yarrowia lipolytica*. Bioresour. Technol. 317:123991. doi: 10.1016/j.biortech.2020.123991, PMID: 32805480 PMC7561614

[ref23] Lou-Bonafonte J. M. Martínez-Beamonte R. Sanclemente T. Surra J. C. Herrera-Marcos L. V. Sanchez-Marco J.. (2018). Current insights into the biological action of Squalene. Mol. Nutr. Food Res. 62:1800136. doi: 10.1002/mnfr.201800136, PMID: 29883523

[ref24] Morabito C. Bournaud C. Maës C. Schuler M. Cigliano R. A. Dellero Y.. (2019). The lipid metabolism in thraustochytrids. Prog. Lipid Res. 76:101007. doi: 10.1016/j.plipres.2019.101007, PMID: 31499096

[ref25] Nagano N. Taoka Y. Honda D. Hayashi M. (2009). Optimization of culture conditions for growth and docosahexaenoic acid production by a marine Thraustochytrid, *Aurantiochytrium limacinum* mh0186. J. Oleo Sci. 58, 623–628. doi: 10.5650/jos.58.623, PMID: 19915319

[ref26] Nakazawa A. Matsuura H. Kose R. Kato S. Honda D. Inouye I.. (2012). Optimization of culture conditions of the thraustochytrid *Aurantiochytrium* sp. strain 18W-13a for squalene production. Bioresour. Technol. 109, 287–291. doi: 10.1016/j.biortech.2011.09.127, PMID: 22023965

[ref27] Pade N. Erdmann S. Enke H. Dethloff F. Dühring U. Georg J.. (2016). Insights into isoprene production using the cyanobacterium *Synechocystis* sp. PCC 6803. Biotechnol. Biofuels 9:89. doi: 10.1186/s13068-016-0503-4, PMID: 27096007 PMC4836186

[ref28] Pade N. Hagemann M. (2014). Salt acclimation of cyanobacteria and their application in biotechnology. Lifestyles 5, 25–49. doi: 10.3390/life5010025, PMID: 25551682 PMC4390839

[ref29] Patel A. Rova U. Christakopoulos P. Matsakas L. (2019). Simultaneous production of DHA and squalene from *Aurantiochytrium* sp. grown on forest biomass hydrolysates. Biotechnol. Biofuels 12:255. doi: 10.1186/s13068-019-1593-6, PMID: 31687043 PMC6820942

[ref30] Patel A. Rova U. Christakopoulos P. Matsakas L. (2020). Mining of squalene as a value-added byproduct from DHA producing marine thraustochytrid cultivated on food waste hydrolysate. Sci. Total Environ. 736:139691. doi: 10.1016/j.scitotenv.2020.139691, PMID: 32497881

[ref31] Rude M. A. Schirmer A. (2009). New microbial fuels: a biotech perspective. Curr. Opin. Microbiol. 12, 274–281. doi: 10.1016/j.mib.2009.04.004, PMID: 19447673

[ref32] Shabala L. McMeekin T. Shabala S. (2009). Osmotic adjustment and requirement for sodium in marine protist thraustochytrid. Environ. Microbiol. 11, 1835–1843. doi: 10.1111/j.1462-2920.2009.01908.x, PMID: 20849566

[ref33] Shabala L. McMeekin T. Shabala S. (2013). Thraustochytrids can be grown in low-salt media without affecting PUFA production. Mar. Biotechnol. 15, 437–444. doi: 10.1007/s10126-013-9499-y, PMID: 23568670

[ref34] Singh D. Barrow C. J. Puri M. Tuli D. K. Mathur A. S. (2016). Combination of calcium and magnesium ions prevents substrate inhibition and promotes biomass and lipid production in thraustochytrids under higher glycerol concentration. Algal Res. 15, 202–209. doi: 10.1016/j.algal.2016.02.024

[ref35] Spanova M. Daum G. (2011). Squalene—biochemistry, molecular biology, process biotechnology, and applications. Eur. J. Lipid Sci. Technol. 113, 1299–1320. doi: 10.1002/ejlt.201100203

[ref36] Wang Q. Ye H. Sen B. Xie Y. He Y. Park S.. (2018). Improved production of docosahexaenoic acid in batch fermentation by newly-isolated strains of *Schizochytrium* sp. and Thraustochytriidae sp. through bioprocess optimization. Synthet. Syst. Biotechnol. 3, 121–129. doi: 10.1016/j.synbio.2018.04.001, PMID: 29900425 PMC5995480

[ref37] Yan N. Marschner P. Cao W. Zuo C. Qin W. (2015). Influence of salinity and water content on soil microorganisms. Int. Soil Water Conserv. Res. 3, 316–323. doi: 10.1016/j.iswcr.2015.11.003

[ref38] Yang C. Gao X. Jiang Y. Sun B. Gao F. Yang S. (2016). Synergy between methylerythritol phosphate pathway and mevalonate pathway for isoprene production in *Escherichia coli*. Metab. Eng. 37, 79–91. doi: 10.1016/j.ymben.2016.05.003, PMID: 27174717

[ref39] Yokochi T. Honda D. Higashihara T. Nakahara T. (1998). Optimization of docosahexaenoic acid production by *Schizochytrium limacinum* SR21. Appl. Microbiol. Biotechnol. 49, 72–76. doi: 10.1007/s002530051139

[ref40] Zhang A. Mernitz K. Wu C. Xiong W. He Y. Wang G.. (2021). ATP drives efficient terpene biosynthesis in marine thraustochytrids. MBio 12:e0088121. doi: 10.1128/mBio.00881-21, PMID: 34182781 PMC8262955

[ref41] Zhang A. Xie Y. He Y. Wang W. Sen B. Wang G. (2019). Bio-based squalene production by *Aurantiochytrium* sp. through optimization of culture conditions, and elucidation of the putative biosynthetic pathway genes. Bioresour. Technol. 287:121415. doi: 10.1016/j.biortech.2019.121415, PMID: 31078814

