# Supplementary material for: Optimal NaCl Medium Enhances Squalene Accumulation in Thraustochytrium sp. ATCC 26185 and Influences the Expression Levels of Key Metabolic Genes
Source: Front Microbiol. 2022 May 4;13:900252. doi: 10.3389/fmicb.2022.900252 (PMC9114700; doi:10.3389/fmicb.2022.900252)
Supplement: Supplementary file 1 [file Data_Sheet_1.docx]

Supplementary Material


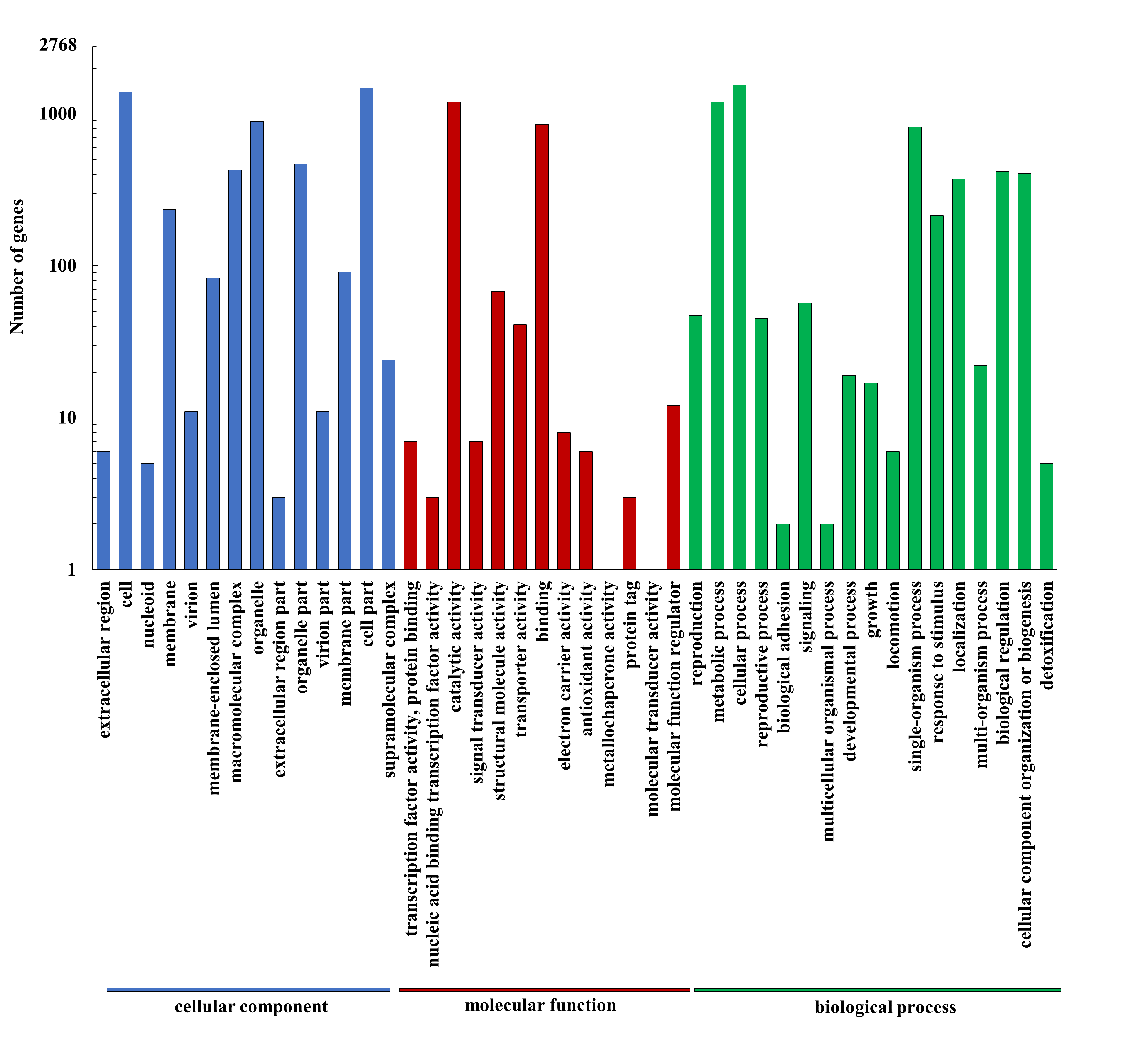


**Figure S1**. Gene ontology (GO) enrichment analysis of ATCC 26185 transcriptome. The columns indicate the number of identified genes. All genes were classified into three groups: cellular component, molecular function, and biological process.

**
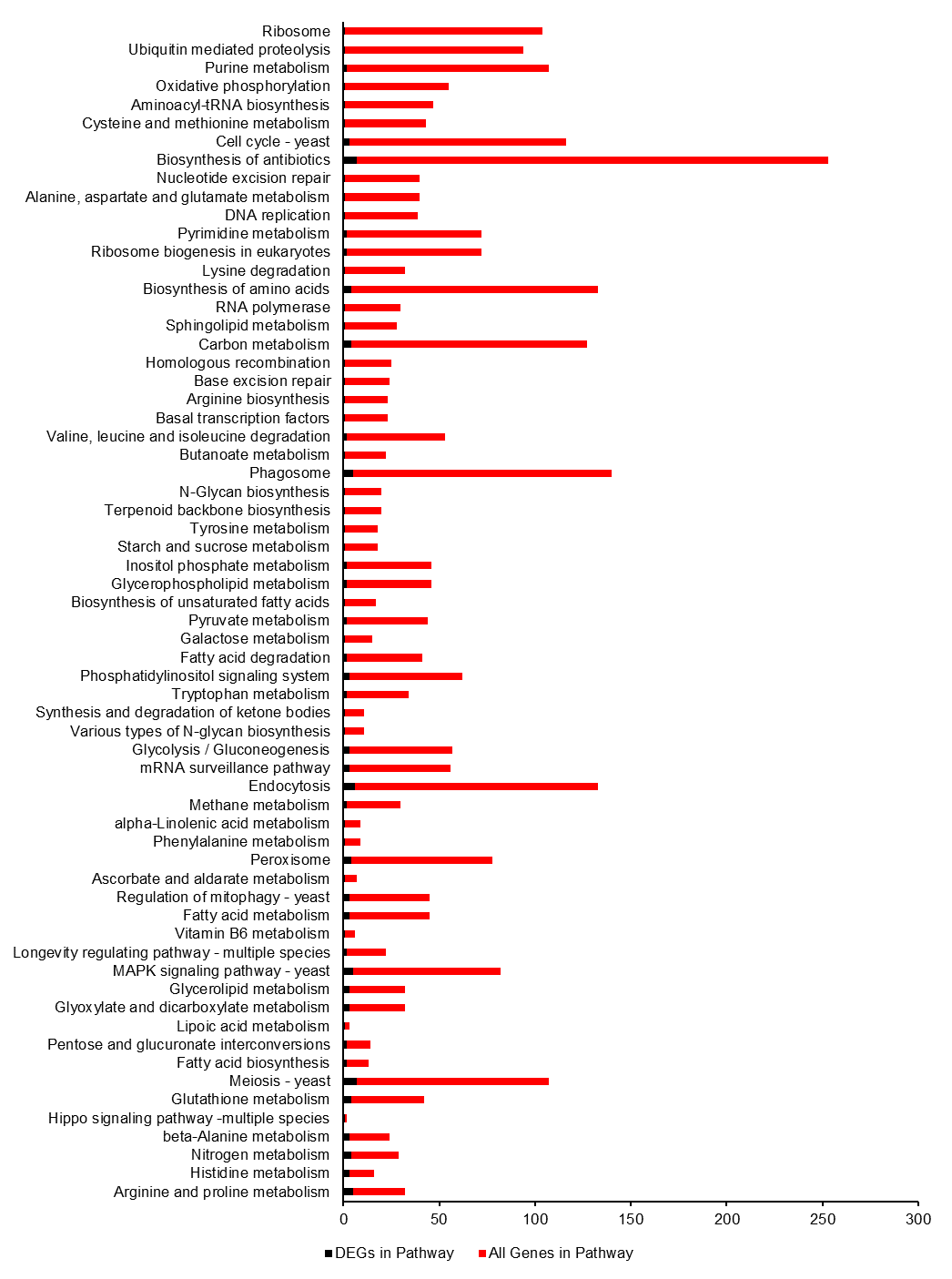
**

**Figure S2**. Different Expression Unigene KEGG Enrichment Analysis. No significantly (FDR ≤ 0.01) enriched KEGG pathways were detected.

**
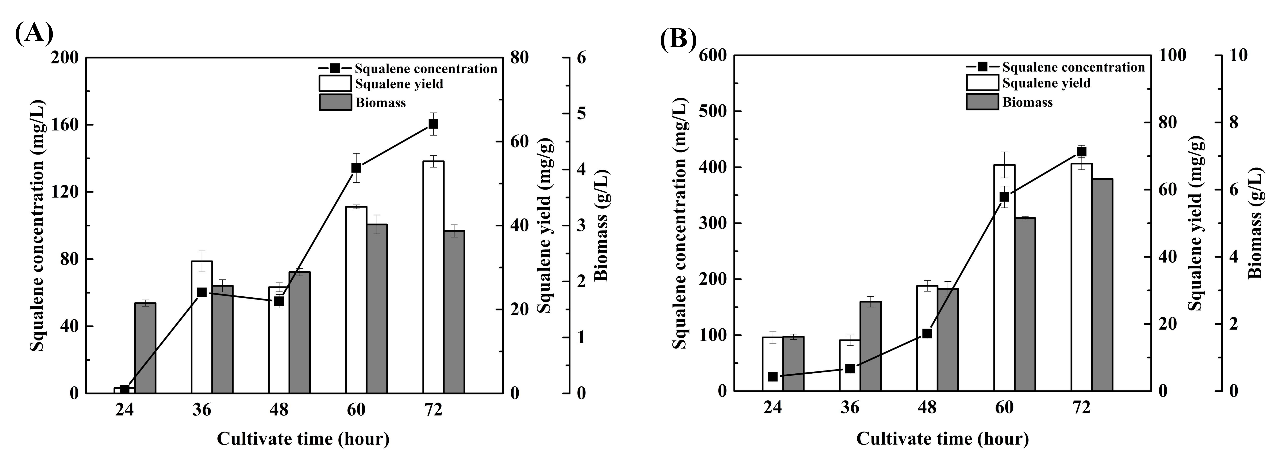
**

**Figure S3**. Time course of biomass, squalene concentration and yield during the cultivation of ATCC 26185 strain in optimal medium containing: (A) 0 g/L NaCl and (B) 5 g/L NaCl.

**Table S1.** Culture conditions for the experiments performed under the OFAT and orthogonal experimental designs.

| Experiment | Variable | Culture condition |
| --- | --- | --- |
| Single-factor experiments | | |
| Growth conditions | Single-factor:  (1) cultivation time:  1 d, 2 d, 3 d, 4 d, 5 d, 6 d, 7 d, 8 d  (2) cultivation temperature:  18 ℃, 23 ℃, 28 ℃, 33 ℃ | SQU medium:30 g glucose, 2 g yeast extract, 2 g monosodium glutamate, 0.2 g (NH_4_)_2_SO4, 0.3 g KH_2_PO_4_, 25 g NaCl, 1 g KCl, 5 g MgSO_4_•7H_2_O, 0.1 g NaHCO_3_, 0.3 g CaCl_2_, 2.9 mg FeCl_3_•6H_2_O, 0.02 mg CuSO_4_•5H_2_O, 0.26 mg CoCl•6H_2_O, 0.6 mg ZnSO_4_•7H_2_O, 8.6 mg MnSO_4_•H_2_O, 1000 mL H_2_O.  Temperature: (1) 28 ℃  Rotating rate: 170 rpm  Seed age: 2 days  Inoculum size: 10%  Cultivation time: 3 days |
| Carbon source | Single-factor:  1. glucose  2. fructose  3. sucrose  4. glycerol  5. lactose  6. corn starch  concentration: 30 g/L  Optimal level:  glucose: 10-50 g/L | Medium: 2 g yeast extract, 2 g monosodium glutamate, 0.2 g (NH_4_)_2_SO_4_, 0.3 g KH_2_PO_4_, 25 g NaCl, 1 g KCl, 5 g MgSO_4_•7H_2_O, 0.1 g NaHCO_3_, 0.3 g CaCl_2_, 2.9mg FeCl_3_•6H_2_O, 0.02 mg CuSO_4_•5H_2_O, 0.26 mg CoCl•6H_2_O, 0.6 mg ZnSO_4_•7H_2_O, 8.6 mg MnSO_4_•H_2_O, 1000 mL H_2_O.  Temperature: 28 ℃  Rotating rate: 170 rpm  Seed age: 2 days  Inoculum size: 10%  Cultivation time: 3 days |
| Nitrogen source | Single-factor:  1. yeast extract  2. tryptone  3. peptone  4. ammonium sulfate  5. sodium nitrate  6. monosodium glutamate (MSG)  7. urea  concentration: 5 g/L  Optimal level:  Yeast extract: 5-30 g/L | Medium: 30 g glucose, 0.3 g KH_2_PO_4_, 25 g NaCl, 1 g KCl, 5 g MgSO_4_•7H_2_O, 0.1 g NaHCO_3_, 0.3 g CaCl_2_, 2.9 mg FeCl_3_•6H_2_O, 0.02 mg CuSO_4_•5H_2_O, 0.26 mg CoCl•6H_2_O, 0.6 mg ZnSO_4_•7H_2_O, 8.6 mg MnSO_4_•H_2_O, 1000 mL H_2_O.  Temperature: 28 ℃  Rotating rate: 170 rpm  Seed age: 2 days  Inoculum size: 10 %  Cultivation time: 3 days |
| NaCl concentration | Concentration:  0-30 g/L | Medium:30 g glucose, 5 g yeast extract, 0.3 g KH_2_PO_4_, 1 g KCl, 5 g MgSO_4_•7H_2_O, 0.1 g NaHCO_3_, 0.3 g CaCl_2_, 2.9 mg FeCl_3_•6H_2_O, 0.02 mg CuSO_4_•5H_2_O, 0.26 mg CoCl•6H_2_O, 0.6 mg ZnSO_4_•7H_2_O, 8.6 mg MnSO_4_•H_2_O, 1000 ml H_2_O.  Temperature:28 ℃  Rotating rate: 170 rpm  Seed age: 2 days  Inoculum size: 10 %  Cultivation time:3 days |
| Orthogonal Experimental Design | | |
| Major factor | glucose:25-35 g/L  yeast extract: 2.5-7.5 g/L  NaCl: 2.5-7.5 g/L | Medium: 0.3 g KH_2_PO_4_, 1 g KCl, 5 g MgSO_4_•7H_2_O, 0.1 g NaHCO_3_, 0.3 g CaCl_2_, 2.9 mg FeCl_3_•6H_2_O, 0.02 mg CuSO_4_•5H_2_O, 0.26 mg CoCl•6H_2_O, 0.6 mg ZnSO_4_•7H_2_O, 8.6 mg MnSO_4_•H_2_O, 1000 mL H_2_O.  Temperature:28 ℃  Rotating rate: 170 rpm  Seed age: 2 days  Inoculum size: 10%  Cultivation time: 3 days |

**Table S2.** Length distribution of assembled transcripts and unigenes.

| Length Range (bp) | Transcripts | Unigenes |
| --- | --- | --- |
| 200-300 | 4848 (15.90 %) | 4298 (18.79 %) |
| 300-500 | 4729 (15.51 %) | 3787 (16.56 %) |
| 500-1000 | 6449 (21.15 %) | 4817 (21.06 %) |
| 1000-2000 | 8027 (26.33 %) | 5695 (24.90 %) |
| >2000 | 6436 (21.11 %) | 4272 (18.68 %) |
| Total Number (bp) | 30,490 | 22,869 |
| Total Length (bp) | 41,489,354 | 28,680,721 |
| N50 Length (bp) | 2140 | 2014 |
| Mean Length (bp) | 1360.75 | 1254.13 |

**Table S3.** Mean value analysis of the orthogonal experimental results.

| Levels | Factors | | |
| --- | --- | --- | --- |
|  | **Glucose** | **Yeast extract** | **NaCl** |
| 1 | 283.38 | 341.49 | 285.81 |
| 2 | 397.42 | 288.62 | 353.62 |
| 3 | 262.18 | 312.89 | 303.56 |

*The values represent the concentration of squalene in mg/L.
